# Retention Throughout the HIV Care and Treatment Cascade: From Diagnosis to Antiretroviral Treatment of Adults and Children Living with HIV—Haiti, 1985–2015

**DOI:** 10.4269/ajtmh.17-0116

**Published:** 2017-10-18

**Authors:** Andrew F. Auld, Valerie Pelletier, Ermane G. Robin, Ray W. Shiraishi, Jacob Dee, Mayer Antoine, Yrvel Desir, Gracia Desforges, Chris Delcher, Nirva Duval, Nadjy Joseph, Kesner Francois, Mark Griswold, Jean Wysler Domercant, Yves Anthony Patrice Joseph, Joelle Deas Van Onacker, Varough Deyde, David W. Lowrance, and the “Groupe d’Analyses SALVH”

**Affiliations:** 1 Division of Global HIV & Tuberculosis, Center for Global Health, Centers for Disease Control and Prevention, Atlanta, Georgia;; 2 Division of Global HIV & Tuberculosis, Center for Global Health, Centers for Disease Control and Prevention, Port au Prince, Haiti;; 3 Programme National de Lutte contre le VIH/SIDA (National AIDS Program), Ministère de la Sante Publique et de la Population (Ministry of Health), Port au Prince, Haiti;; 4 National Alliance of State and Territorial AIDS Directors (NASTAD), Port-au-Prince, Haiti;; 5 Department of Health Outcomes and Policy, University of Florida, Gainesville, Florida;; 6 National Alliance of State and Territorial AIDS Directors (NASTAD), Washington, District of Columbia

## Abstract

Monitoring retention of people living with HIV (PLHIV) in the HIV care and treatment cascade is essential to guide program strategy and evaluate progress toward globally-endorsed 90–90–90 targets (i.e., 90% of PLHIV diagnosed, 81% on sustained antiretroviral therapy (ART), and 73% virally suppressed). We describe national retention from diagnosis throughout the cascade for patients receiving HIV services in Haiti during 1985–2015, with a focus on those receiving HIV services during 2008–2015. Among the 266,256 newly diagnosed PLHIV during 1985–2015, 49% were linked-to-care, 30% started ART, and 18% were retained on ART by the time of database closure. Similarly, among the 192,187 newly diagnosed HIV-positive patients during 2008–2015, 50% were linked to care, 31% started ART, and 19% were retained on ART by the time of database closure. Most patients (90–92%) at all cascade steps were adults (≥ 15 years old), among whom the majority (60–61%) were female. During 2008–2015, outcomes varied significantly across 42 administrative districts (arrondissements) of residence; cumulative linkage-to-care ranged from 23% to 69%, cumulative ART initiation among care enrollees ranged from 2% to 80%, and cumulative ART retention among ART enrollees ranged from 30% to 88%. Compared with adults, children had lower cumulative incidence of ART initiation among care enrollees (64% versus 47%) and lower cumulative retention among ART enrollees (64% versus 50%). Cumulative linkage-to-care was low and should be prioritized for improvement. Variations in outcomes by arrondissement and between adults and children require further investigation and programmatic response.

## INTRODUCTION

Haiti has a population of about 11 million people, including 150,000 people living with HIV (PLHIV) and outside of sub-Saharan Africa, it is one of the countries worst affected by the global HIV pandemic.^1^ Despite having significant resource limitations and being ranked 163rd out of 186 countries on the Human Development Index,^2^ with only about 2.5 doctors per 10,000 population,^3^ Haiti has often led the global HIV pandemic response among low- and middle-income countries (LMIC).^4^ For example, a 2005 report of successful outcomes for the first 1,004 patients receiving antiretroviral therapy (ART) in Port-au-Prince served as a demonstration project that providing ART for PLHIV in resource-constrained settings could be feasible and effective. In addition, in 2010, Haiti was the first country to show that earlier ART initiation at CD4 < 350 cells/µL resulted in superior treatment outcomes compared with delaying ART until CD4 fell to ≤ 200 cells/µL.^5,6^ Despite political instability^7^ and natural disasters,^8^ by 2016, Haiti had enrolled more than 70,000 PLHIV on ART, which is an important global health achievement.^1^

To facilitate continued scale-up of high-quality care nationwide, in 2008, the Ministry of Health (MOH), with support from the Centers for Disease Control and Prevention (CDC), the National Alliance of State and Territorial AIDS Directors, and other partners initiated a project to create a nationally representative, longitudinal, patient monitoring system.^9^ This national monitoring system is called the haitian active longitudinal tracking of HIV system, with the French acronym being SALVH. Formerly SALVH was known as the HIV/AIDS surveillance system.^9^ SALVH captures patient-level data throughout the clinical care continuum from the point of HIV diagnosis onward.^9^

In this initial descriptive analysis of SALVH data, we aim to describe 1) key historical components of Haiti’s HIV testing, care, and treatment programs and 2) the HIV care and treatment cascade from diagnosis through retention on ART, stratified by key covariates including age group, sex, year of service uptake, and arrondissement of residence. Prior peer-reviewed publications of the HIV care continuum in Haiti have been limited to individual implementing partners, which were often affiliated with universities in the United States and may have had higher per-patient fiscal and human resources to invest in services.^5,6,10–12^ In contrast, SALVH provides nationally representative HIV care continuum data. Therefore, lessons learned from this analysis can inform national strategy as Haiti aims to reach globally endorsed 90–90–90 targets (90% of PLHIV diagnosed, 90% of those diagnosed on sustained ART, and 90% of those on sustained ART with viral suppression) before 2020.^13^

## MATERIALS AND METHODS

### Haiti’s HIV testing and treatment guidelines.

Haiti’s HIV testing and treatment guidelines have changed over time (Table 1).^14^ In early phases of the HIV epidemic response between the late 1980s and 2002, HIV testing and treatment services were offered to patients at specialized health centers based on perceived clinical need and availability of diagnostic tests and antiretrovirals by the MOH and a limited number of non-Governmental Organizations, including the Haitian Group for the Study of Kaposi’s Sarcoma and Opportunistic Infections and partners in health.^14^ Stand-alone voluntary counseling and testing services have been scaled up since 2002. Haiti adopted World Health Organization (WHO)-recommended provider-initiated testing and counseling guidelines in 2007.^15^ Community-based testing services (home-based testing and mobile testing services) have been scaled up since 2011.

**Table 1:** Evolution of Haiti’s HIV testing, linkage-to-care, and treatment guidelines over time—1985–2016

	Before 2008*	2008–Nov/2010	Dec/2010–Nov/2013	Dec/2013–June 2016	July 2016–Present
HIV testing and linkage to care guidelines					
–	HIV testing services offered to patients based on clinical presentation since 1990. Stand-alone voluntary counseling and testing (VCT) services scaled up since 2001. Provider-initiated testing and counseling services offered for all persons presenting for healthcare services since 2007 in high risk settings. Community-based testing services (home-based testing and mobile testing) scaled-up since 2011.				
Adult ART eligibility criteria (≥ 15 years old)					
CD4 count criteria	CD4 ≤ 200	CD4 ≤ 200, consider if 200–350	CD4 ≤ 350	CD4 ≤ 500	Treat all
WHO stage criteria	WHO stage IV	WHO stage III/IV	WHO stage III/IV	WHO stage III/IV	
Pediatric ART eligibility criteria (< 15 years old)					
Age criteria	–	–	Treat all children < 1 years old†	Treat all children < 2 years.‡ Treat all children < 5 years since November 2015	Treat all
CD4% criteria	< 25% (< 12 months old)	< 25% (< 12 months old)	< 25% (12–59 months old)**	< 25% (≥ 24 months old)††	
	< 15% (≥ 12 months old)*	< 20% (12–59 months old)‡‡			
	–	< 15% (≥ 60 months old)			
CD4 count criteria	< 1,500/µL (< 12 months)***	< 750/µL (12–35 months)‡‡	< 750/µL (24–59 months)**	< 750/µL (24–59 months)††	
	< 750/µL (12–35 months)	< 350/µL (36–59 months)	< 350/µL (≥ 60 months old)	< 350/µL (≥ 60 months old)	
	< 350/µL (36–59 months)	< 200/µL (≥ 60 months old)	–	–	
	< 200/µL (≥ 60 months old)	–	–	–	
WHO stage criteria	WHO stage III/IV	WHO stage III/IV	WHO stage III/IV	WHO stage III/IV	
Special populations ART-eligibility criteria					
HIV/TB coinfection	–	–	Irrespective of CD4	Irrespective of CD4	Treat all
Pregnant women	–	–	Option B+ since 2012 (treat all)	Option B+ (treat all)	
HIV/Hepatitis B coinfection	–	–	Treat all	Treat all	
Serodiscordant couples	–	–	–	Treat all	
Key populations	–	–	–	–	
Advanced age	–	–	Treat all > 60 years old	Treat all > 50 years old	
HIV nephropathy	–	–	Treat all	Treat all	
ART monitoring†††					
Clinical visit schedule	1, 2, 3, and 6 m, quarterly	1, 2, 3, and 6 m, quarterly	1, 2, 3, and 6 m, quarterly	1, 2, 3, and 6 m, quarterly	1, 2, 3, and 6 m, quarterly
ART medication pick-up schedule	Monthly	Monthly	Monthly	Monthly	Monthly
CD4	Every 6 months	Every 6 months	Every 6 months	Every 6 months	As deemed necessary
Viral load	–	–	VL in cases of suspected treatment failure	VL in cases of suspected treatment failure	VL at 6 and 12 months after initiation then every 12 months in stable patients
Pre-ART monitoring†††					
Co-trimoxazole pick-up schedule	Monthly	Monthly	Monthly	Monthly	Monthly during ART
CD4	Every 6 months	Every 6 months	Every 6 months	Every 6 months	CD4 recommended at ART initiation

ART = antiretroviral therapy; WHO = World Health Organization; TB = tuberculosis; VL = viral load; m = month.

Note: Unless referenced by a footnote, all data presented in the table reflects guidelines provided by the Haiti HIV treatment program team in preparation of this article.

* Reference: George E, Noel F, Bois G, Cassagnol R, Estavien L, Rouzier Pde M, Verdier RI, Johnson WD, Pape JW, Fitzgerald DW, Wright PF, 2007. Antiretroviral therapy for HIV-1-infected children in Haiti. *J Infect Dis 195:* 1411–1418. Note that at the time, 2006 WHO pediatric guidelines were to initiate if CD4% was < 25% (< 12 months old), < 20% (12–35 months old), or < 15% (≥ 36 months old).

† Reference: Program data used by in-country teams for planning HIV programs. Note that from 2010 onwards, WHO recommended that all children < 2 years old be eligible for ART.

‡ Reference: Program data used by in-country teams for planning HIV programs. Note that from 2013 onwards, WHO recommended that all children < 5 years old be eligible for ART and since 2015 WHO has recommended that all children, regardless of age, CD4, or WHO stage, start ART.

** 2010 WHO Guidelines: World Health Organization. Antiretroviral therapy for HIV infection in infants and children: toward universal access—Recommendations for a public health approach—2010 revision. Available at: http://apps.who.int/medicinedocs/documents/s18809en/s18809en.pdf. Accessed September 1, 2016.

†† Reference: MOH: Directives Nationales Pour les Soins et le Traitement des Nourrissons, des Enfants et des Adolescents Exposes au VIH ou Porteurs du Virus (2013). Available at: http://www.hivpolicywatch.org/duremaps/data/guidelines/HaitiPaediatricARTguidelines2013.pdf. Accessed September 1, 2016.

‡‡ 2008 WHO Guidelines: Report of the WHO Technical Reference Group, Pediatric HIV/ART Care Guideline Group Meeting WHO Headquarters, Geneva, Switzerland, 10–11 April 2008. Available at: http://www.who.int/hiv/pub/paediatric/WHO_Paediatric_ART_guideline_rev_mreport_2008.pdf. Accessed September 1, 2016.

*** 2006 WHO Guidelines, 2006. Antiretroviral Therapy of HIV Infection in Infants and Children: Towards Universal Access: Recommendations for a public health approach. Available at: http://www.who.int/hiv/pub/guidelines/paediatric020907.pdf.

††† There are some variations in clinic visit schedules between clinics. Some non-Governmental organizations support provision of directly observed treatment of ART. Monthly medication pick-ups are not needed at all clinics, especially for stable patients. Availability of both CD4 testing and viral load testing varies by clinic.

Before 2008, only patients with a CD4+ T-cell (CD4) count ≤ 200/µL or WHO stage IV were ART-eligible. Since 2008, Haiti’s ART-eligibility guidelines evolved over four key phases: January 2008 to November 2010,^16^ December 2010 to November 2013, December 2013 to June 2016, and July 2016 to present.^17,18^ During these phases, ART eligibility guidelines have become increasingly inclusive (Table 1). For example, CD4 thresholds of ART eligibility for adults living with HIV increased from ≤ 200/µL in phase 1, to ≤ 350/µL in phase 2, ≤ 500/µL in phase 3, and test-and-start (i.e., universal ART eligibility)^19^ in phase 4. In addition, since 2012, all HIV-positive pregnant women have been eligible for life-long ART through the prevention of mother-to-child transmission program Option B+ (referred to as Option B+ in this article).^20^ Similarly, CD4%, CD4 count, and age criteria for ART eligibility have become more inclusive for children over time (Table 1).

For HIV-positive patients newly registering for care and determined to be initially ART ineligible, biannual clinic visits and CD4 monitoring have been recommended since the start of Haiti’s HIV treatment program. At ART initiation, monthly for 3 months, and then quarterly, patients attend clinician check-ups during which standardized MOH-recommended records are completed. Most patients collect medications monthly from clinic pharmacies. For all patients late for monthly ART pick-up appointments, text messaging followed by telephonic tracing and, if necessary, home visits are recommended, but resource-limitations have limited coverage of these activities.

### Study design and population.

This was an observational cohort study analyzing patient-level data in SALVH.^9^ SALVH is a uniquely comprehensive patient monitoring system that links patient-level data, captured at the time of HIV diagnosis, with corresponding electronic medical records (EMRs)^21^ maintained at health facilities providing HIV care and treatment services.^9^ At both the HIV testing locations and treatment facilities, trained data clerks prospectively enter patient data into electronic systems with real-time consistency and acceptable value range checks helping to maintain data quality. By June 2016, patient data from 224 testing and treatment facilities were available for analysis, representing approximately 90% of public HIV service providers, including 100% of the United States government-funded care providers in the country. Since 2014, data captured in electronic systems at HIV testing locations and treatment facilities have been routinely transferred to a central data warehouse, comanaged by MOH and other stakeholders, for further quality checks, dataset concatenation, deduplication, and data management.^9^

For this analysis, all adult and pediatric medical records in SALVH were eligible for analysis of the care and treatment continuum from HIV diagnosis onward to allow fuller description of Haiti’s HIV epidemic response. However, because more recent data collected since 2008^8^ are of higher relevance to current program strategy, when analyzing outcome determinants following HIV diagnosis, care enrolment, and ART initiation, respectively, the analysis is restricted to those newly HIV diagnosed, newly enrolled in care, and newly starting ART since 2008. For these recent new patients, the HIV care and treatment cascades are stratified by age, gender, year of service uptake, and arrondissement of residence. Facility-level databases were closed for analysis by June 2016; 198 (88%) of 224 site-level databases, representing 86% of patients were closed in the first 6 months of 2016.

### Clinical care cascade outcomes.

Outcomes of each of the following three key cascade steps were evaluated: 1) from diagnosis to enrollment in care, 2) from enrollment in care to ART initiation, and 3) from ART initiation onward. Enrollment in care was defined as the first evidence of care registration at a health facility, co-trimoxazole prescription, or CD4 testing. Loss to follow-up (LTFU) at any stage along the care continuum was defined as no contact with HIV clinical services in the 12 months preceding the time point the database was closed for this analysis (referred to as database closure in this article). As previously reported, the SALVH system complies with CDC recommendations for reducing the number of duplicate case reports to the system under 5%.^9^

The outcomes of interest following HIV diagnosis were as follows: 1) documented death before care enrollment, 2) LTFU before care enrollment, 3) enrollment in care, and 4) retention in the cascade step, which represents a category of patients who have been diagnosed with HIV in the 12 months before database closure, received their result, and referred to a healthcare facility, but have not yet reached the healthcare facility.

The outcomes of interest following HIV care enrollment were as follows: 1) documented death before ART start, 2) LTFU before ART start, 3) ART initiation, and 4) retention in pre-ART care (i.e., alive and in pre-ART care at the time of database closure).

The outcomes of interest following ART initiation were documented death, LTFU, and overall cumulative retention (i.e., alive and on ART at the time of database closure).

### Stratification variables.

HIV care and treatment cascades were stratified by age, gender, year of HIV service uptake, and arrondissement of residence (with arrondissement being the second subnational administrative level in Haiti, similar to a “district” in other countries). The arrondissements used in this analysis are the same as those used by MOH and president’s emergency plan for AIDS relief (PEPFAR) in planning annual Country Operational Plans. When comparing cascade step outcomes between annual cohorts, only patients who started each cascade step more than 12 months before database closure were included so that all patients in the analysis had the potential to meet the LTFU definition. Patients < 15 years of age were considered children while patients ≥ 15 years of age were considered adults for this report.^22^

### Analytic methods.

Data were analyzed using the STATA 13 (StataCorp, Stata Statistical Software, Release 13, College Station, TX).

Patients with missing date of HIV diagnosis (*N* = 7) were excluded from the analysis. Among patients included in the analysis, complete data were available for time-to-event analysis (i.e., complete data on the date services began and date of outcome were available). Missing data for the stratification variables are described and the cascade analysis restricted to those patients where the data needed for disaggregation were present. This was a descriptive analysis with continuous variables summarized with medians and interquartile ranges (IQR) and categorical variables summarized using frequencies and percentages.

### Ethics approval.

Use of routine anonymized data for this study was approved by the Haitian National Ethics Committee and was designated as nonresearch by the Center for Global Health at the CDC.

## RESULTS

### Program history.

Overall, following deduplication, 266,256 patients with recorded dates of HIV diagnosis were captured in the SALVH system. Among these newly diagnosed HIV-positive patients, 131,542 (49%) were subsequently documented to enroll in HIV care, while 78,917 (30%) started ART.

**Figure 1.**
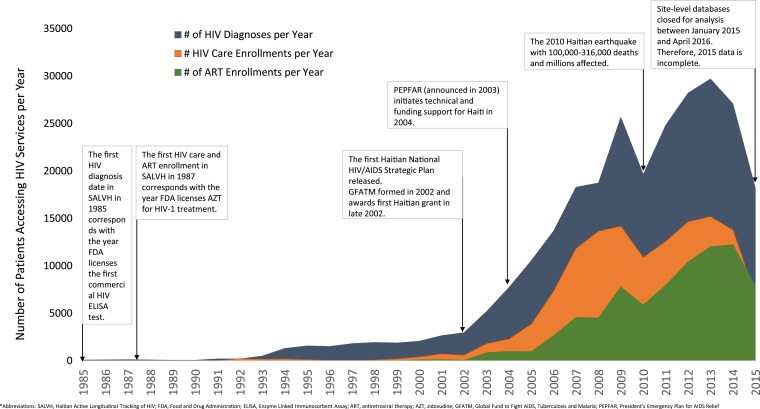
Number of new HIV diagnoses, enrollments in care, and ART enrollments per year—Haiti, 1985–2015.

The earliest documented HIV diagnosis in SALVH was in 1985, which is 2 years after the first AIDS patient was reported from Haiti in 1983. The earliest care enrollment and ART enrollment was in 1987 (Figure 1). Between 1985 and 2002, the annual rate of new HIV diagnoses, care enrollments, and ART enrollments remained low at ≤ 2,955/year, ≤ 581/year, and ≤ 120/year, respectively (Figure 1). However, in 2003 compared with 2002, annual rates of new HIV diagnoses, care enrollments, and ART enrollments were 1.8, 3.1, and 18.8 times as high following the release of the first Haitian National HIV/AIDS Strategic Plan and the first Haitian grant from the Global Fund to Fight AIDS, tuberculosis (TB), and Malaria (GFATM) in late 2002. In addition, compared with 2003, annual rates of new HIV diagnoses, care enrollments, and ART enrollments were 1.5, 1.3, and 1.2 times as high in 2004, following initiation of PEPFAR support in 2004. Rates of new diagnoses, care enrollments, and ART enrollments, continued to increase annually during 2004–2009, a time of increasing support from PEPFAR and GFATM.

However, in 2010, compared with 2009, there was a notable drop in annual HIV diagnoses, care enrollments, and ART enrollments of 23%, 23%, and 25%, respectively, corresponding with the 2010 Haitian earthquake. Between 2011 and 2013, annual new diagnoses, care enrollments, and ART enrollments accelerated again. The highest annual rate of new HIV diagnoses was in 2013 (29,696/year) and corresponded with the highest annual rate of new care enrollments (15,194/year). The highest rate of new ART enrollments was in 2014 (12,223/year). Significant deceleration in 2015 corresponds with proximity to the time of database closure for analysis.

### Baseline cohort characteristics.

Among the 266,256 patients at HIV diagnosis, 80% had available age data and 96% data on gender. The frequency of missing age at diagnosis was somewhat higher among those diagnosed before 2004 (4,543 [24%] of 18,922) than among those diagnosed from 2004 onward (48,521 [20%] of 247,334). Among patients with age data available, 193,948 (91%) were adults (≥ 15 years old) and 19,244 (9%) were children (< 15 years old) (Table 2). Among adults, 61% were female and median age was 33 (IQR 26–41). Among children, 52% were female and median age was < 1 year (IQR 0–3).

**Table 2:** Patient characteristics at HIV diagnosis, care enrollment, and ART enrollment—1985–2015

	At HIV diagnosis			At HIV care enrollment			At ART enrollment		
	*n*	*N*	%/media*n* (IQR)	*n*	*N*	%/median (IQR)	*n*	*N*	%/median (IQR)
Sex									
Female	155,315	254,685	61%	79,468	130,226	61%	48,391	78,439	62%
Male	99,370	254,685	39%	50,758	130,226	39%	30,048	78,439	38%
Missing	11,571	266,256	4%	1,316	131,542	1%	478	78,917	1%
Sex adults (≥ 15 years old)									
Female	118,423	193,948	61%	61,355	100,456	61%	38,391	62,346	62%
Male	75,525	193,948	39%	39,101	100,456	39%	23,955	62,346	38%
Sex children (< 15 years old)									
Female	10,011	19,244	52%	5,483	10,662	51%	2,645	5,181	51%
Male	9,233	19,244	48%	5,179	10,662	49%	2,536	5,181	49%
Age in years, median (IQR)	–	213,192	32 (24–40)	–	111,118	33 (25–42)	–	67,527	35 (27–43)
Missing	53,064	266,256	20%	20,424	131,542	16%	11,390	78,917	14%
Adult age in years, median (IQR)		193,948	33 (26–41)	–	100,456	34 (27–43)	–	62,346	36 (29–44)
Child age in years, median (IQR)		19,244	0 (0–3)	–	10,662	0 (0–4)	–	5,181	2 (0–7)
CD4 count, median (IQR)	NA	NA	NA	–	74,010	350/µL (164–564)	–	54,281	235/µL (110–359)
Missing	–	–	–	57,532	131,542	44%	24,636	78,917	31%
Adult CD4 count, median (IQR)	NA	NA	NA	–	59,525	343/µL (162–555)	–	45,091	234/µL (110–353)
Missing	–	–	–	40,931	100,456	41%	17,255	62,346	28%
Child CD4 count, median (IQR)	NA	NA	NA	–	3,015	601/µL (300–979)	–	2,222	392/µL (187–756)
Missing	–	–	–	7,647	10,662	72%	2,959	5,181	57%

NA = not available; IQR = interquartile range; ART = antiretroviral therapy.

Among the 131,542 care enrollees, 84% had age data and 99% had gender data. Among patients with age data available, 100,456 (90%) were adults and 10,662 (10%) were children (Table 2). Among adults, 61% were female, median age was 34 (27–43), and median CD4 count was 343/µL (IQR 162–555). Among children, 51% were female, median age < 1 year (IQR 0–4), and median CD4 count was 601 (IQR 300–979).

Among the 78,917 ART enrollees, 86% had age data and 99% gender data. Among patients with age data available, 62,346 (92%) were adults and 5,181 (8%) were children (Table 2). Among adults, 62% were female, median age was 35 (IQR 27–43), and median CD4 count was 234/µL (IQR 110–353). Among children, 51% were female, median age was 2 (IQR 0–7), and median CD4 count was 392/µL (IQR 187–756).

### National HIV care and treatment cascades.

**Figure 2.**
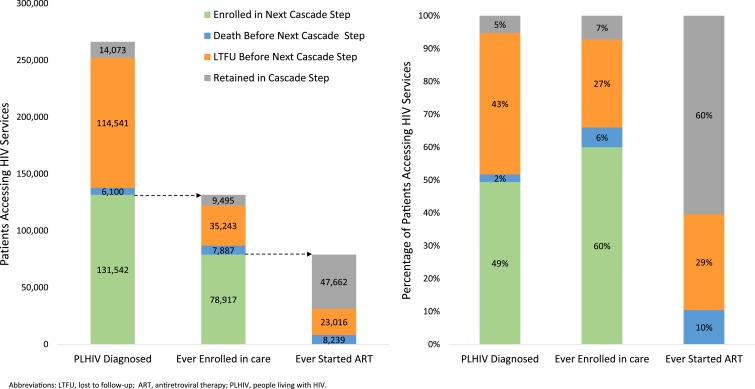
HIV care and treatment cascade—Haiti, 1985–2015.

**Figure 3.**
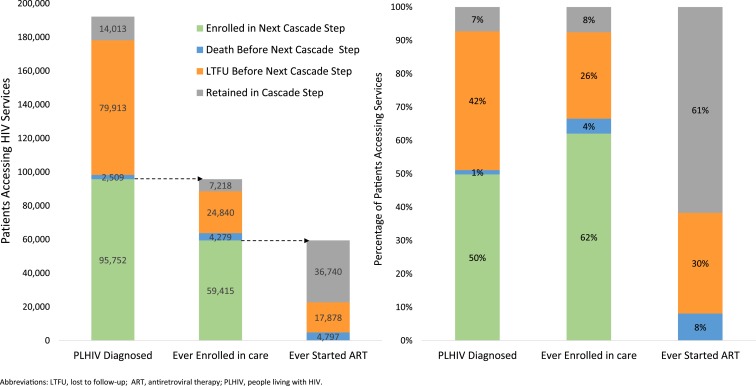
HIV care and treatment cascade—Haiti, 2008–2015.

Among the 266,256 patients diagnosed HIV-positive during 1985–2015, 49% were linked-to-care, 2% were documented to have died before linkage-to-care, 43% were LTFU before linkage-to-care, and 5% were retained in the cascade step, by the time of database closure (Figure 2). Among the 131,542 care enrollees, 60% had started ART, 6% died during pre-ART care, 27% were LTFU during pre-ART care, and 7% were alive in pre-ART care by database closure. Among the 78,917 ART enrollees, 10% had died, 29% were LTFU, and 60% were retained on ART by database closure. National cascade outcomes for the 192,187 (72%) of 266,256 patients diagnosed HIV-positive since 2008 were similar to those diagnosed positive during 1985–2015 (Figure 3).

### Stratification by sex, age, and year of service uptake.

**Figure 4.**
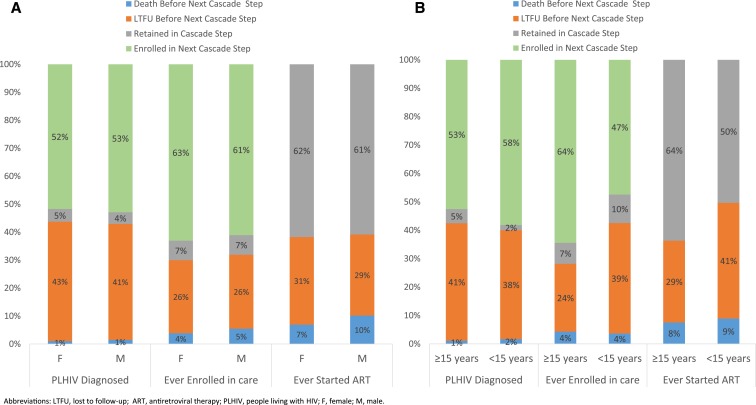
HIV care and treatment cascade by gender (**A**) and age (**B**)—Haiti, 2008–2015.

Linkage-to-care following HIV diagnosis was similar between males (53%) and females (52%) diagnosed during 2008–2015 (Figure 4). Similarly, the percentage of care enrollees starting ART by database closure was similar between males (61%) and females (63%), and the percentage of ART enrollees retained on ART was similar between males (61%) and females (62%).

However, there were cascade outcome differences between adults and children (Figure 4). During 2008–2015, although linkage-to-care was similar for adults (53%) and children (58%), the percentage of care enrollees who started ART by database closure was lower among children than adults (47% versus 64%), and the percentage of ART enrollees retained at database closure was lower among children than adults (50% versus 64%).

**Figure 5.**
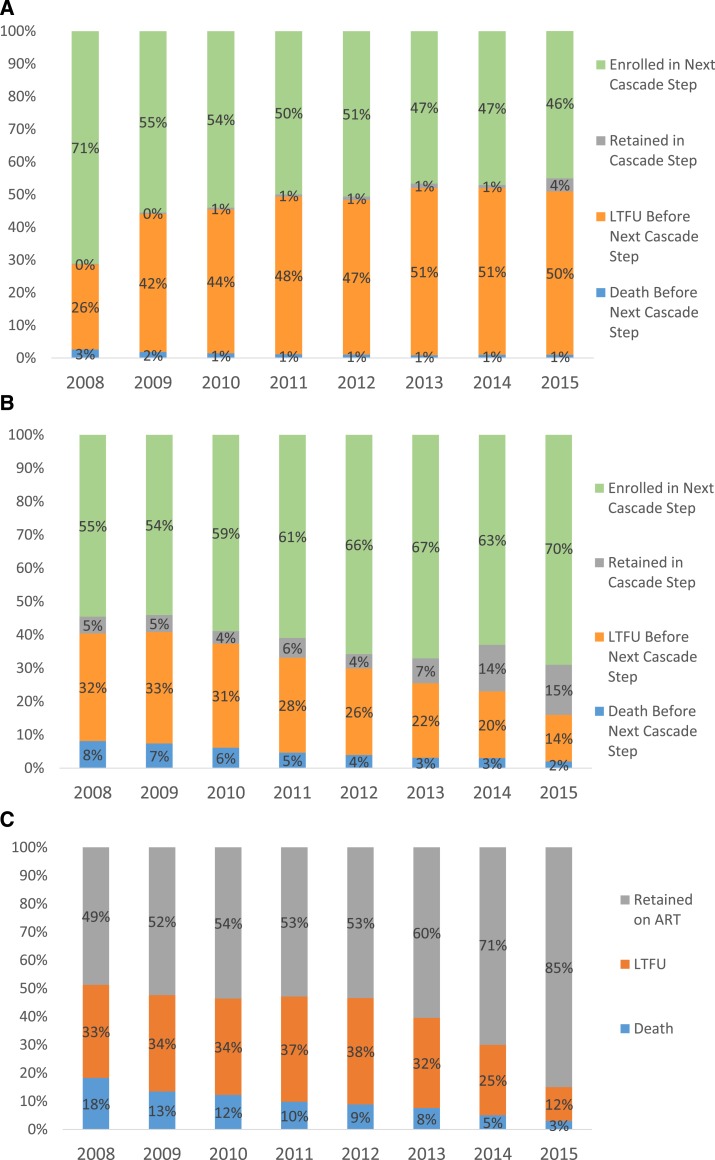
Outcomes of (**A**) HIV diagnosis by year of diagnosis, (**B**) care enrollment by year of care enrollment, and (**C**) ART enrollment, by year of ART enrollment—Haiti, 2008–2015.

Among patients diagnosed more than 12 months before database closure, linkage-to-care by time of database closure was lower for those diagnosed more recently than those diagnosed in 2008 (Figure 5A). However, the proportion of care enrollees starting ART by database closure was higher in recent years compared with 2008; 70% of care enrollees in 2015 versus 55% of 2008 care enrollees had started ART by database closure (Figure 5B). More recent ART enrollees were more likely to be retained on ART by time of database closure than 2008 ART enrollees (Figure 5C).

### Stratification by arrondissement of residence at diagnosis.

**Figure 6.**
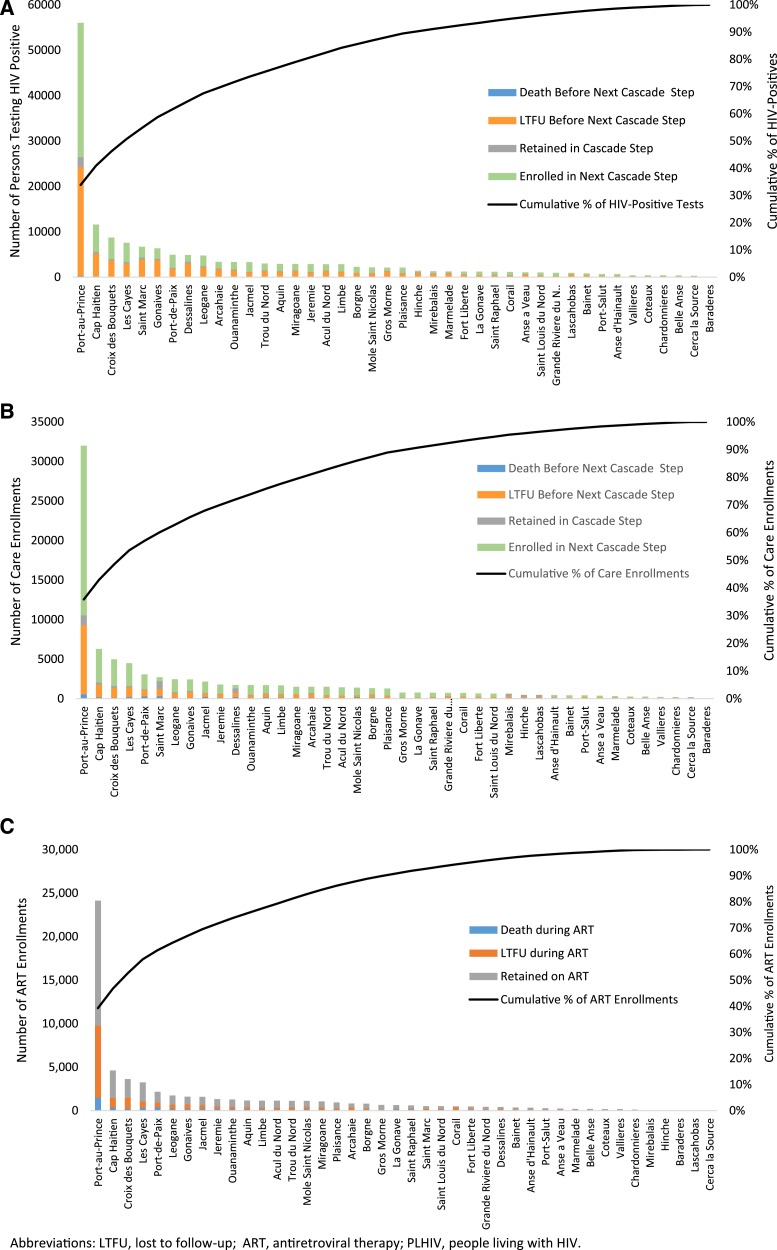
Numbers of (**A**) HIV-positive diagnoses, (**B**) care Enrollments, and (**C**) ART Enrollments by Arrondissement of Patient Residence—Haiti, 2008–2015.

Figure 6A and B show that about 80% of new HIV diagnoses and new care enrollees since 2008 were among residents of 16 (38%) of 42 arrondissements. Figure 6C shows that about 80% of ART enrollees were among residents of 14 (33%) of 42 arrondissements. The largest arrondissement (Port au Prince), which is home to about 24% of Haiti’s total population, accounted for 34% of new HIV diagnoses, 36% of care enrollments, and 39% of ART enrollments.

**Figure 7.**
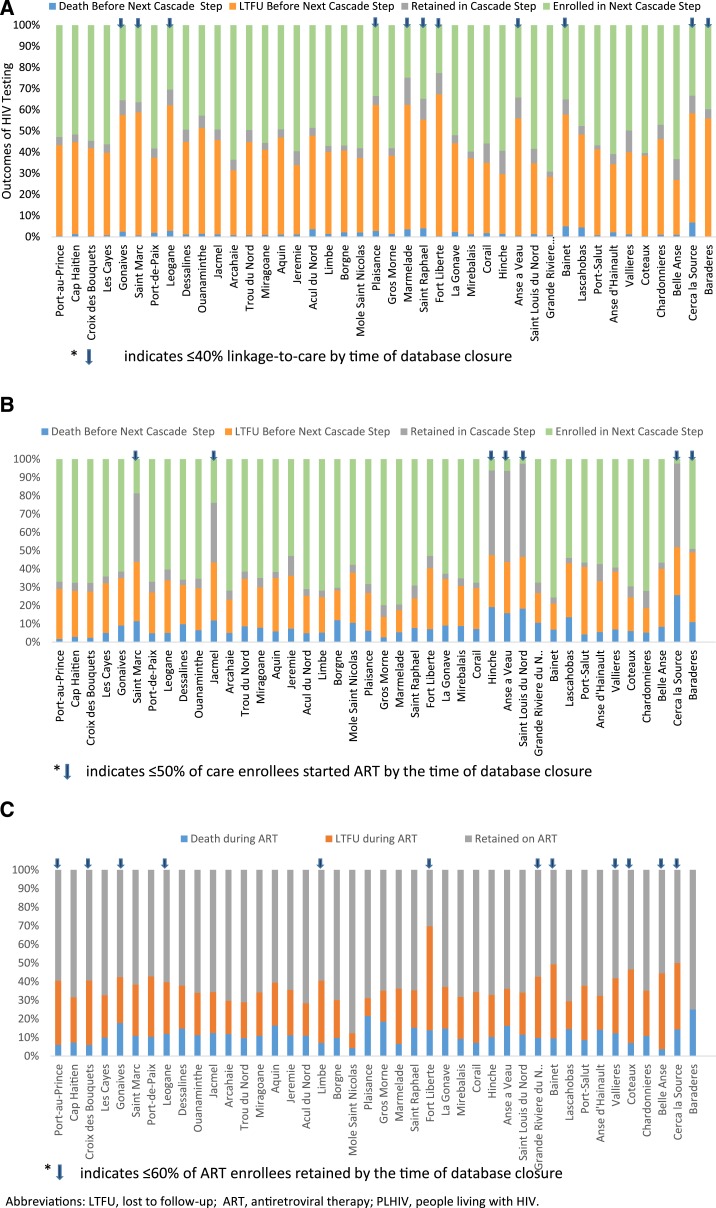
Outcomes of (**A**) HIV-positive diagnoses, (**B**) care enrollments, and (**C**) ART Enrollments by Arrondissement of Patient Residence—Haiti, 2008–2015.

Figure 7 shows that outcomes following HIV diagnosis, care enrollment, and ART enrollment varied by arrondissement. In Figure 7A, blue arrows indicate the 11 (26%) of 42 arrondissements with ≤ 40% overall linkage-to-care success among newly diagnosed HIV-positive patients since 2008. Overall, linkage-to-care success by the time of database closure ranged from 23% to 69% across arrondissements. Figure 7B shows the 7 (17%) of 42 arrondissements where ≤ 50% of care enrollees were started on ART by the time of database closure. Overall, the percentage of 2008–2015 care enrollees who started ART by the time of database closure ranged from 2% to 80% across arrondissements. Figure 7C shows the 12 (29%) of 42 arrondissements where ≤ 60% of ART enrollees since 2008 were retained on ART by database closure. Overall, the percentage of 2008–2015 ART enrollees retained by database closure ranged from 30% to 88% across arrondissements.

## DISCUSSION

Few monitoring systems in LMIC are able to capture longitudinal patient-level data from the time of HIV diagnosis through retention on ART and none, to our knowledge, are able to capture these data for ≥ 90% of PLHIV accessing HIV services nationally.^23^ Therefore, this first comprehensive description of Haiti’s HIV care and treatment cascade using the SALVH system, covering 30 years of epidemic response, and representing over 200,000 patients, is unique and has several important findings that can inform national strategy for reaching 90–90–90 targets.

These data show the successes of the national HIV response and help to illustrate the importance of the national commitment and GFATM and PEPFAR support since 2002–2004. Although the peak of Haiti’s epidemic was in the late 1990s with annual numbers of new HIV infections peaking at about 32,000 new infections per year in 1996,^1^ it was only following GFATM support in late 2002 and PEPFAR support in 2004 that significant accelerations in HIV diagnosis, care enrollment, and treatment were observed. The acceleration in diagnosing and treating PLHIV corresponds with accelerations in donor assistance for health from about $33.3 million in 2002 to $64.5 million in 2003, $77.1 million in 2004, $90.6 million in 2005, and reaching $282.6 million in 2013.^24^ According to estimates from the Joint United Nations Program on HIV/AIDS (UNAIDS), by the end of 2015, of the 150,000 PLHIV, about 54% are estimated to know their status up from < 3% in 2002, and about 46% were receiving ART up from < 2% in 2002.^1^ Over the same time period (2002–2015), the annual number of new HIV infections has fallen from about 11,000/year to 8,300/year.^1^ Given the strong evidence that ART reduces HIV transmission risk within serodiscordant partnerships^25^ and the increases in ART coverage are associated with reductions in population-level HIV incidence,^26^ the sustained acceleration in ART uptake since 2002–2004 has almost certainly contributed to improved epidemic control.^27,28^

Due to the 7.0 magnitude earthquake in January 2010, which resulted in an estimated 200,000 deaths, 1.5 million internally displaced persons,^29^ and significant disruptions to the health system,^30^ rates of new HIV diagnoses, HIV care enrollments, and ART initiations were about 25% lower in 2010 compared with 2009. While previous reports have documented the immediate postearthquake recovery in rates of service uptake in the months following the earthquake,^8,31,32^ this report provides additional data that 1) the recovery was sustained longer term, with annual increases in rates of service uptake through the end of 2013, and 2), the postearthquake recovery efforts allowed service uptake rates during 2012–2014 to exceed pre-earthquake levels. Given the extent of the earthquake damage and uncertainty about longer term impact on HIV service uptake at the time of the earthquake, these data are encouraging for program managers and funders and can help inform HIV program response following natural disasters in other LMIC.^8^

The low cumulative linkage-to-care incidence by database closure among patients diagnosed during 2008–2015 (50%) is lower than that reported from a meta-analysis of program data from sub-Saharan Africa (SSA) for newly diagnosed adult patients during 2006–2011 (59%)^23^ and much lower than success reported from a research-oriented nongovernmental organization in Haiti for newly diagnosed adult patients during 2012–2014 (81%).^33^ While cumulative incidence percentages are less amenable to comparison with other programs than 3-, 6-, and 12-month linkage-to-care estimates,^34^ these findings suggest, similar to SSA, there is needed improvement in linkage-to-care programs nationally in Haiti.

In this analysis, the main determinants of cumulative linkage-to-care success by database closure were year of HIV diagnosis and arrondissement of residence. Those diagnosed in 2008 had better linkage-to-care success (71%) than those diagnosed during 2009–2015 (46–55%). Although this is at least partly due to the fact that recently diagnosed patients have had less time to achieve linkage than patients diagnosed in earlier years, the drop-off in cumulative linkage-to-care success percentages between patients diagnosed in 2008 and patients diagnosed during 2009–2015 warrants some attention. Further exploration of trends over time using 3-, 6-, and 12-month linkage estimates stratified by the year of diagnosis is needed.^35^

The wide variation in linkage-to-care success rates by arrondissement of residence requires further investigation. Known barriers to linkage-to-care include distance from the clinic,^36^ lack of friendly services for patients,^37^ and over-crowded clinics,^38^ and these barriers might vary by arrondissement. In addition, with about 76% of the population living on < $2.5 per day, poverty remains an important barrier to accessing health services for most PLHIV in Haiti.^39^ Interventions to improve linkage-to-care include improving clinic operations to remove barriers to ART initiation^40^ and ART initiation on the same day of HIV diagnosis for eligible patients.^33,41^ Targeting the 11 arrondissements with ≤ 40% linkage-to-care success rates with facility and service provision assessments and improvement interventions might be one approach to efficiently target resources to locations most in need of additional support. However, the ≤ 40% threshold was chosen as an example to indicate the approximately 25% of arrondissements with the lowest linkage-to-care cumulative incidence percentages by database closure; the MOH would need to decide on the criteria for geographic focus as well as the thresholds involved based on perceived need for programmatic change and the availability of financial and human resources.^42,43^ Investing in interventions to improve linkage-to-care could yield significant additional benefit in reducing HIV incidence. For example, in the U.S., it is estimated that 61% of new HIV transmissions are from patients with known HIV status but not linked to care.^44^

Similar to a recent report from Haiti,^10^ the median adult CD4 count at HIV care enrollment was quite high (343/µL, IQR 162–555) during 2008–2015, higher than the overall median CD4 count at care enrollment for four SSA countries during 2006–2011 (242–292/μL),^45^ and higher than median CD4 count at care enrollment reported from a recent meta-analysis of SSA programs (about 251/μL).^46^ Because more than 25% of adults enrolling in care have a CD4 count > 500/µL, this suggests Haiti could see a significant acceleration in ART enrollment with the recently adopted Treat All guidelines as patients, who are retained in pre-ART care with a CD4 > 500/µL, transition onto ART.^41,47^ However, impact of Treat All guideline adoption in July 2016 on rates of ART enrollment still needs to be evaluated.

The percentage of care enrollees during 2008–2015 who started ART by database closure (62%) is higher than reports from most SSA programs.^23^ The SSA meta-analysis reported 46% retention from care enrollment to ART eligibility and 68% retention from ART eligibility to ART initiation^23^; if we multiply these two percentage values, this suggests that only 31% of care enrollees in SSA during 2006–2011 subsequently started ART.^23^ Similarly, a recent evaluation involving 390,603 HIV care enrollees from Kenya, Mozambique, Rwanda, and Tanzania reported that 30% of care enrollees during 2005–2011 started ART by database closure in 2012.^35^

In this analysis, the key determinants of ART initiation following care enrollment were age (adult versus child), year of care enrollment, and arrondissement of residence. Several factors might explain poorer outcomes among children enrolling in care compared with adults. Firstly, among perinatally infected children, HIV disease progression in the absence of ART is faster than that observed for adults, with median time to death being about 2 years in children versus 11 years in adults.^48^ Secondly, among children in early adolescence, rates of LTFU are known to be higher than among adults.^49–51^ The adoption of the Treat All strategy in Haiti, should increase the percentage of children starting ART soon after care enrollment, which is a program priority.^19,41,47^

The increasing cumulative incidence of ART initiation after care enrollment by database closure for 2015 versus 2008 care enrollees (70% versus 55%) is probably explained by increasingly inclusive ART eligibility guidelines for adults and children (see Table 1), which is encouraging for program managers. Care enrollment outcomes varied by arrondissement of residence even more widely than linkage-to-care outcomes, with incidence of ART initiation after care enrollment by database closure ranging from 2% to 80%. Fact-finding assessments and interventions in the seven arrondissements where ≤ 50% of care enrollees had started ART by database closure might be an efficient use of limited resources.^43^

Median CD4 count at ART enrollment among adults (234/µL) was higher than that reported from a recent meta-analysis of ART programs in SSA (152/µL).^46^ Early adoption of universal treatment of pregnant women since 2012 might be one reason for the relatively high CD4 at ART initiation.^22^ Evaluation of trends over time and stratification by male, female, and pregnant female could inform which populations Haiti is reaching early with ART, and which populations are harder to reach with early ART.^22^

LTFU after ART initiation accounted for most (73%) of attrition among ART enrollees during 2008–2011. While a certain percentage of patients LTFU from ART may have re-engaged with care at a different facility, between 20% and 60% of patients LTFU, are likely to have died by the time of database closure, usually shortly after missing their last scheduled appointment.^52^ Therefore, the LTFU percentage for enrollees during 2008–2015 (30%) is an overestimate and the mortality incidence (10%), an underestimate.^53^ Recent analyses show that risk of death increases 20-fold for patients who default from ART.^54^ As such, LTFU should be regarded as an important factor on the causal pathway to patient mortality and should be prioritized for intervention.^54^

In this analysis, age, year of enrollment, and arrondissement of residence were associated with cumulative retention by database closure. Lower ART retention among children might be related to several challenges associated with treating children with ART. For example, children are more likely to have faster disease progression, adherence challenges, suboptimal ART dosing because of failure to increase antiretroviral medication dosages as the patient grows,^55^ higher rates of LTFU (especially among adolescents),^49^ and higher background mortality rates (especially among young children < 5 years old) compared with adults receiving ART.^56^ Recent adoption of Treat All guidelines for children might reduce overall attrition rates.^19^ In addition, additional training in providing pediatric ART might be warranted to improve outcomes.^55^

Improvements in cumulative retention by database closure for 2015 versus 2008 ART enrollees (85% versus 49%) is at least partly related to the fact that more recent ART enrollees have had less time to experience death and LTFU outcomes; evaluation of trends in 6- and 12-month ART retention is warranted to explore these trends, but is beyond the scope of this initial analysis.^22^ Variations in cumulative ART retention by database closure by arrondissement of residence from 30% to 88% suggest there may be local factors affecting attrition (LTFU or death) rates.^42^ There are many causes for LTFU, including difficulties affording transport to the clinic,^57^ work and childcare responsibilities,^57^ overcrowded clinics,^58,59^ and long wait times due to increased patient-to-provider ratios, with shortages of pharmacists or pharmacy assistants being a common cause for long wait times.^22,58^ Therefore, LTFU prevention interventions may need to be tailored to the arrondissement in question to address main drivers of LTFU by arrondissement.^54^ The most common cause of death among PLHIV, including ART enrollees, in LMIC is TB,^60^ accounting for about 40% of deaths. In about half of all TB-related deaths, TB remains undiagnosed and untreated *antemortem*.^60^ Early accurate diagnosis and treatment of active TB^59,60^ or exclusion of active TB to allow prescription of isoniazid preventive therapy are needed to reduce TB-related mortality.^61,62^

A key factor influencing both rates of enrollment and outcomes at all stages of the cascade was arrondissement of residence. This finding has two important implications. Firstly, with 75% to 90% of HIV program funding coming from international donors^63,64^ and plateaued international funding since 2009, Haiti’s HIV care and treatment program operates in an increasingly resource-constrained environment. Given these constraints, PEPFAR is encouraging HIV programs to focus resources on subnational units with the highest burden of HIV to maximize impact.^64,65^ In the case of Haiti, it is estimated that about a quarter of all PLHIV reside in Port au Prince and about 84% reside in 20 of 42 arrondissements. Consequently, the MOH and partners are prioritizing resources to the highest burden arrondissements to maximize impact.^64,65^ Secondly, the variations in outcomes by arrondissement of residence highlight important opportunities to focus quality improvement (QI) initiatives; arrondissements with the highest absolute burden of patients seeking testing and treatment services, the highest cumulative incidence of attrition (death or LTFU) from the HIV care continuum, and the highest absolute number of patients lost through attrition should be prioritized for QI activities.^42,43,66^

This article has several strengths and limitations. Strengths include the large sample size, generalizability of findings for the national program, duration of observation (30 years), and ability to describe the continuum of HIV care from diagnosis onward. An important limitation is that cumulative incidence estimates of the various outcomes by the time of database closure are presented; presenting time-specific incidence percentages (e.g., 3-, 6-, or 12-month outcome incidence percentages), or using incidence rates (e.g., rates per 100 person years) are needed to best facilitate comparisons (e.g., comparison between cohorts enrolled in different calendar years, or comparisons between Haiti outcomes and other national outcomes), and these analyses are planned for separate articles. In addition, caution comparing outcomes by arrondissement of residence is warranted because there might be variations between arrondissement in the time periods covered because of variations in the date that HIV testing, care, or treatment services started in each arrondissement and variations in the timing of site-level database closures by arrondissement. Other limitations include missing covariate data, as is common when using routine programmatic data systems, and missing final vital status for enrolled patients who became LTFU. Because a large proportion of LTFU patients are likely to have died by database closure,^52^ the LTFU percentages are likely over-estimates and mortality percentages under-estimates.^53^ Ideally a tracing study should be implemented to improve mortality estimates.^67^ Existing model-based approaches for improving ART mortality estimates will be appropriate when 12-month ART mortality estimates are presented in future analyses.^22,68^ Although there have been significant investments in improving data quality throughout SALVH,^9^ primary data validation comparing paper records at participating sites with corresponding electronic records has been limited because of human and fiscal resource constraints. In addition, this is a descriptive analysis, with no attempt to identify covariate effect size using crude and adjusted regression analyses, which will be presented separately.

## CONCLUSION

Encouraging findings from this initial descriptive analysis of SALVH data include the sustained acceleration in HIV service provision postearthquake, the clear impact of GFATM and PEPFAR on HIV service uptake rates, the relatively high median CD4 counts at both pre-ART care and ART enrollment, and the relatively high cumulative incidence of ART initiation after care enrollment compared with similar LMIC in SSA. The analysis also highlights key challenges and additional needed analyses. Key challenges include the relatively low linkage-to-care success compared with similar LMIC in SSA, poorer care and ART outcomes among children compared with adults, and significant variations in outcomes throughout the HIV care continuum by arrondissement of residence. Further research to explore trends in patient characteristics and outcomes over time, reasons for variations in outcomes by arrondissement, and patient- and site-level predictors of outcomes, is needed.
